# Smartphone MEMS Accelerometer and Gyroscope Measurement Errors: Laboratory Testing and Analysis of the Effects on Positioning Performance

**DOI:** 10.3390/s23177609

**Published:** 2023-09-01

**Authors:** Vincenzo Capuano, Liangchun Xu, Jose Estrada Benavides

**Affiliations:** Samsung Semiconductor, Inc., San Jose, CA 95134, USA; liangchun.xu@samsung.com (L.X.); ji.estrada@samsung.com (J.E.B.)

**Keywords:** MEMS, INS, navigation, positioning, smartphones

## Abstract

Embedding various sensors with powerful computing and storage capabilities in a small communication device, smartphones have become a prominent platform for navigation. With the increasing popularity of Apple CarPlay and Android Auto, smartphones are quickly replacing built-in automotive navigation solutions. On the other hand, smartphones are equipped with low-performance Micro Electro Mechanical Systems (MEMS) sensors to enhance their navigation performance in Global Navigation Satellite System (GNSS)-degraded or -denied environments. Compared with higher-grade inertial navigation systems (INS), MEMS-based INSs have a poor navigation performance due to large measurement errors. In this paper, we present laboratory test results on the stochastic and deterministic errors observed in MEMS inertial sensor measurements of five different smartphones from different manufacturers. Then, we describe and discuss the short-term effects of these errors on the pure inertial navigation performance and also on the navigation performance based on the tight coupling of INS with GNSS measurements using a smartphone.

## 1. Introduction

Smartphones are equipped with a variety of sensors that can measure their position and orientation. With powerful computing and storage capabilities, smartphones have become a popular platform for pedestrian and automotive navigation. For pedestrian navigation, a smartphone is usually an obvious option. For automotive navigation, with the increasing popularity of Apple CarPlay and Android Auto, smartphones are quickly replacing built-in navigation solutions. Navigation capabilities mainly rely on processing GNSS signals for outdoor positioning. However, in environments where GNSS signals are degraded or denied, such as urban canyons, tunnels, and parking garages, smartphones estimate their position and orientation using inertial sensors. This allows them to navigate regardless of any surrounding obstacles that may obstruct the GNSS signals. By using an INS composed of a three-axis accelerometer and a gyroscope, it is possible to determine the position and orientation of a smartphone and the vehicle hosting it. The achievable positioning performance strongly depends on the accuracy, stability, and quality of the inertial sensors.

The accelerometers, gyroscopes, and magnetometers used in a smartphone are typically very small and lightweight MEMS (Micro Electro Mechanical System) sensors [1], very inexpensive, but generally characterized by a poor performance [2,3]. An example of MEMS specifications is provided in [4,5].

While there are a number of literature sources on the testing and validation of inertial sensors, such as [6,7,8,9,10,11], to the best of the authors’ knowledge, there are not many works that specifically focus on evaluating the navigation performances achievable with the MEMS inertial sensors of a smartphone. The only work is [12], in which inertial measurements were collected with a Sony Xperia M2 Aqua and Samsung Galaxy Note 3 in three different driving scenarios and processed offline to determine position.

In the first part of this paper, we present the main stochastic and deterministic error characteristics of the MEMS sensors embedded in five smartphone devices, a *Google Pixel 7 Pro*, a *Samsung SM-A536V*, a *Vivo X60 Pro*, an *iPhone XR*, and *One Plus 7 Pro*, determined by the means of laboratory tests. Identifying the characteristics of these errors is crucial for designing a navigation filter. It helps to define the state and measurement components, formulate system and measurement models, and tune the filter parameters, such as the system and measurement covariance. In the second part, we analyze the effects of these errors on the positioning accuracy when using a smartphone. For such a goal, we discuss the short-term accuracy achievable in: (1) a straight-line motion with pure MEMS inertial mechanization, by converting the MEMS accelerometer and gyro measurements into position, velocity, and attitude; and (2) a real drive trajectory around our campus in San Jose, CA, with an INS-GNSS tight fusion.

The paper is organized as follows. Section 2 summarizes the error sources that characterize an inertial measurement. In Section 3, we describe the three main tests carried out in our laboratory for each of the five considered smartphones and present their results: the *Single-Position Static Test* to determine the stochastic error of the accelerometers and gyro; the *Six-Position Static Test* to estimate the deterministic error of the accelerometers; and the *Gyro Rate Test* to determine the deterministic errors of the gyros. In Section 4, for the five considered devices, we estimate the short-term achievable positioning accuracy when using INS measurements affected by the MEMS errors determined in Section 3. In Section 5, we discuss the obtained results. The conclusions are drawn in Section 6.

## 2. Error Characteristics of MEMS Inertial Sensors

The works [13,14,15,16,17,18] provide a comprehensive overview of the various sources of errors affecting inertial measurements. The typical value of each of these errors can widely vary depending on the class or grade of the inertial sensor. There is no universally agreed definition of low-, medium, and high-grade inertial sensors. A common classification, provided in [13], includes five main broad categories from the highest grade to the lowest: the Marine, Aviation, Intermediate, Tactical, and Consumer grades. The lowest categories exhibit much larger errors than the highest categories, but, at the same time, they are characterized by a smaller mass, volume, power consumption, and are usually drastically cheaper (less than $10 for consumer-grade versus more than $1M for marine-grade). The current research on the development of inertial sensors is primarily focused on MEMS technology that enables the low-cost mass production of very small, light quartz and silicon sensors. MEMS are generally the smallest and cheapest inertial sensors, however, the lowest consumer-grade MEMS sensors, usually adopted in a smartphone, offer a poor performance. The final performance of a MEMS inertial sensor is strongly influenced by whether or not the sensor has been calibrated; often, MEMS sensors are sold without any lab calibration as consumer-grade, and with calibration as tactical-grade.

Multiple accelerometers and gyros are integrated into an inertial navigation unit (IMU) and their measurements are processed together in an INS to produce a navigation solution.

In general, all grades of accelerometers and gyros manifest biases, scale factor, cross-coupling errors, and random noise. Depending on the type of sensor, they can also exhibit higher-order errors and angular rate specific force cross-sensitivity [13]. They are also affected by vibration-induced errors [19].

We can distinguish *deterministic* errors and *stochastic* errors for MEMS sensors. Deterministic errors include constant and proportional errors to the measuring values of the sensor. Specifically for an inertial sensor, there are four individual components of a deterministic error: (1) a fixed contribution which can be corrected by sensor calibration; (2) a temperature-dependent variation while the sensor is working out of the range of its normal operating temperature; (3) a run-to-run variation which changes from different runs but stays constant during one run; and (4) last but not least, in-run variation which slowly changes within the course of a run.

We generally refer to accelerometer and gyro *bias* as a constant error, independent of the specific force and angular rate, respectively. These usually are the main contribution to the overall position error. It is possible to differentiate static bias from dynamic bias. Static bias corresponds to the fixed and run-to-run contribution of a deterministic error. Depending on the type of sensor, dynamic bias is generally about 10% of the static one [13] and it includes the in-run, also known as bias instability, and the temperature-dependent error contribution. According to [20], this bias instability varies over time with a period of one or two minutes for low-cost INSs.

The *scale factor* is a departure of the input–output gradient of the instrument from unity following unit conversion by the IMU [13]. Therefore, a scale factor error is proportional to the true measured value. Misalignments of the sensitive axes of the INS with respect to the orthogonal axes of the body frame that result from a non-perfect packaging process are usually known as *cross-coupling errors*. Uncalibrated, consumer-grade MEMS can have scale factors and cross-coupling as large as 0.1 and 0.02, respectively [13].

Stochastic errors or *random noise* affect all inertial sensors due a number of sources [17]. These can be well described by a random probability distribution. It is possible to describe accelerometer and gyro random noise through their integral over time, known as *random walks*. The standard deviation of a random walk will indeed be proportional to the square root of the integration time.

In addition, further error sources are the *quantization* of the sensor data, *g-dependent bias* (depending on the sensor type), *scale factor non linearity*, cross-coupling error variations (*anisoinertia* errors) [21] as a function of the specific force and angular rate, errors due to the exceeding of the operating ranges and bandwidth, and higher-order systematic errors that depend on the type of sensor.

Finally, smartphone operating systems can introduce additional errors, such as increased noise and quantization, inconsistent update rates, data lags, and repeated measurements. For this reason, when designing an INS-based navigation filter for smartphones, it is important to characterize the error of the IMU sensors through laboratory tests, rather than relying solely on the manufacturer’s specifications.

## 3. Laboratory Tests and Results

For this study, we conducted laboratory tests to determine the main error sources and particularly the standard deviation, bias instability, random walk and rate random walk, bias, and scale factor affecting the measurements of the accelerometers and gyroscopes integrated in five different smartphones. Table 1 shows the smartphones we tested and the models of their accelerometer and gyroscope sensors. Each smartphone has a three-axis accelerometer and a three-axis gyro. We used the *Single-Position Static Test* to determine the stochastic errors of the accelerometers and gyros. We also used the *Six-Position Static Test* and *Gyro Rate Test* to estimate their deterministic errors. This section provides an overview of our testing methods and results.

### 3.1. Single-Position Static Test

In this test, data from the stationary accelerometers and gyros were collected for four hours to observe the standard deviation of the noisy outputs. An Allan Variance analysis was carried out to quantify the bias instability, random walk, and rate random walk.

The Allan Variance (AVAR) is a time domain analysis technique conceived to characterize the noise and stability in clock systems [22,23]. It can also be applied to analyze the measurement error characteristics of any instrument. AVAR represents the root mean square (RMS) random drift error as a function of the average time τ. By performing specific operations on the data, AVAR can be used to characterize different types of noise in inertial sensor data [24]. The AVAR can be computed as follows, as presented in [11,25,26].

**Figure 1:**
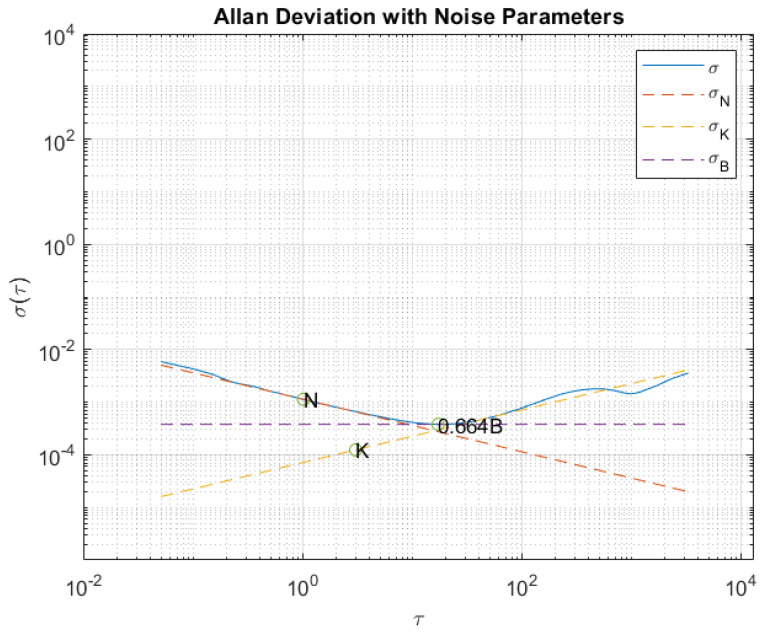
Example of Allan Variance plot for the *y*-axis accelerometer of iPhone XR.

**Figure 2:**
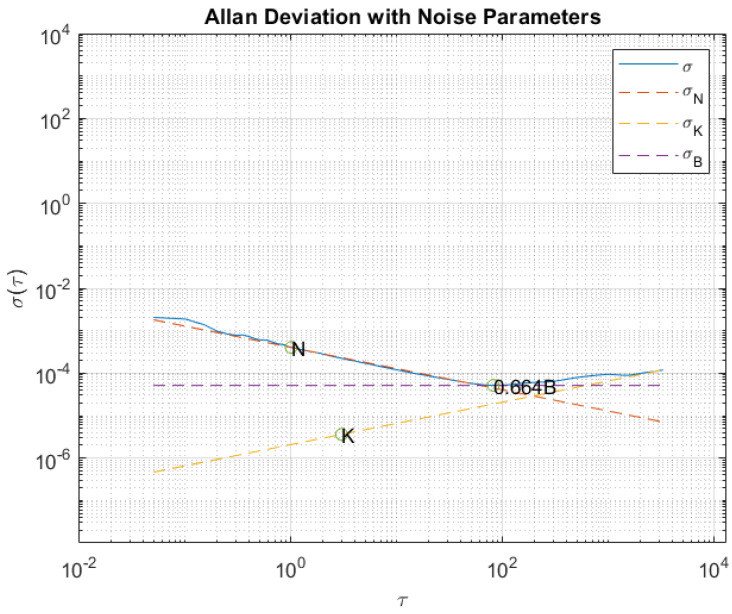
Example of Allan Variance plot for the *y*-axis gyro of iPhone XR.

From the slopes of the Allan deviation ($\sigma =\sqrt{\sigma^{2}}$) log–log plot versus τ, it is possible to extract several noise parameters. Table 2 reports the ones we determined in our analysis for each of the axes (x,y,z) of both the accelerometers and gyroscopes, and in particular: the standard deviation (SD), bias instability (B), angular random walk (N), and rate random walk (K). Figure 1 and Figure 2 show an example of an Allan variance plot for one accelerometer and one gyro, respectively, of the iPhone XR.

Table 3 reports the standard deviation (*SD*) values determined for all five considered devices. The maximum and minimum values across all five smartphones are bolded. We can see that the iPhone XR MEMS measurements had the largest gyro noise SD, with a three-axis average of 0.0027 rad/s, while the Samsung SM-A536V ones had the largest accelerometer SD with a value of of 0.0106 m/s^2^. The One Plus 7 Pro MEMS gyro and accelerometer measurements had the lowest SD instead, with a three-axis average of 0.0042 m/s^2^ and 2.7073 × 10^−4^ rad/s. Compared with the specifications from the manufacturers, the Samsung and Pixel phones were very close to the numbers reported in the data sheet. However, the Vivo and One Plus phones had less noise than the values in the specification sheets.

Table 4 and Table 5 report the bias instability, random walk, and rate random walk for the accelerometer and gyro, respectively, of the five considered devices. The largest accelerometer bias instability and random walks were found for the Samsung SM-A536V. The Google Pixel 7 Pro, Vivo X60 Pro, and iPhone XR showed the lowest accelerometer bias, velocity random walk, and acceleration random walk, respectively. The Google Pixel 7 Pro also appeared to have the lowest gyro bias instability and random walks. The Vivo X60 Pro showed the largest gyro bias instability, while the iPhone XR had the largest velocity and acceleration random walks.

### 3.2. Six-Position Static Test

Usually, the calibration of an IMU is performed using a mechanical platform, which rotates the sensors into different precisely controlled orientations and at known rotational velocities [7,8]. At each orientation and during the rotation, the outputs of the accelerometers and gyros are observed and compared with the pre-calculated gravity force and rotational velocity, respectively.

**Figure 3:**
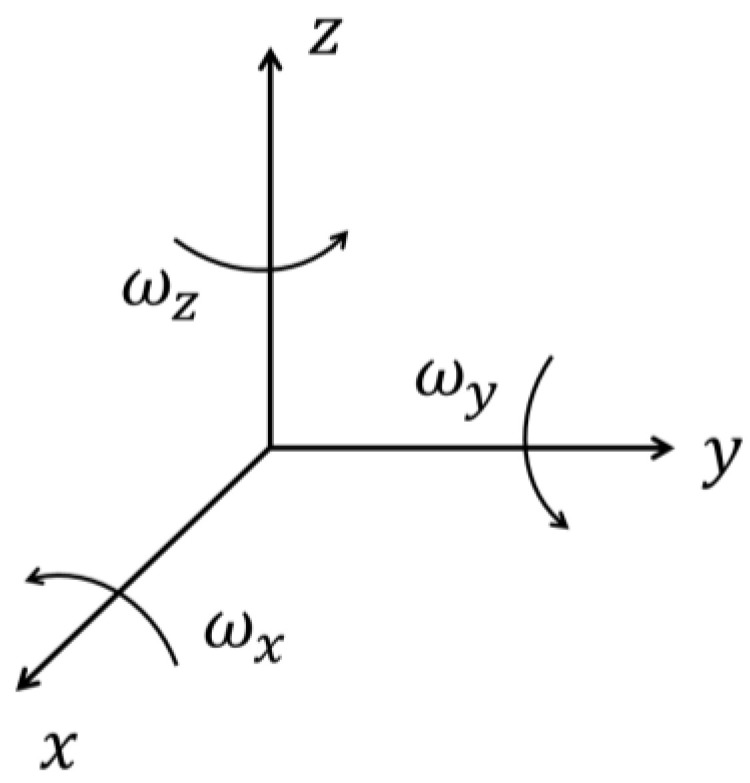
IMU axes.

**Figure 4:**
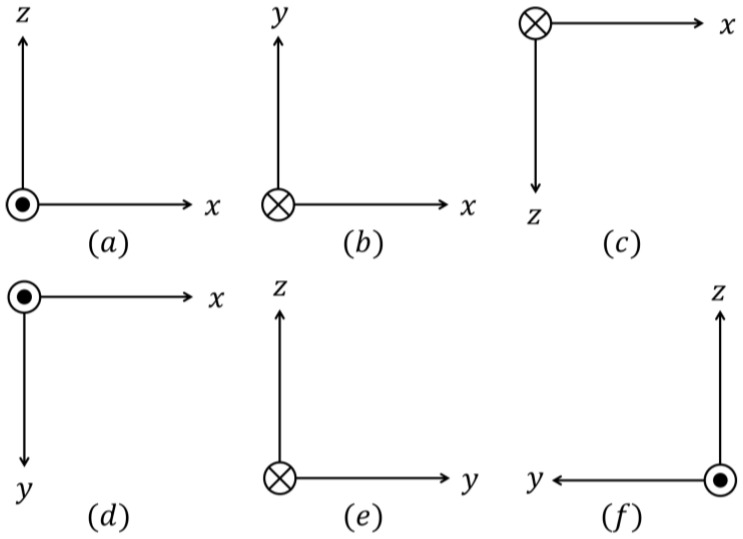
Six positions (**a**–**f**). The six positions of the IMU are indicated by the directions of each orthogonal axis of the IMU. The reference direction corresponds to a vector pointing towards the reader, represented by a dot within a circle. A vector pointing in the opposite direction away from the reader is represented by a cross within a circle.

The *Six-Position Static* is a widely used calibration method [9]. It involves mounting the IMU on a level surface and aligning each sensitivity axis of each sensor illustrated in Figure 3, with the direction of gravity, and its opposite, as shown in Figure 4. Gravity and Earth rotation rate can be the references for the estimation of the bias and scale factor of the accelerometers and gyros, respectively. Note that more than six positions can be considered (multi-position static test); in this case, for the additional positions (orientations), the reference value will be a fraction of the gravity force for the accelerometers and a fraction of the Earth rotation rate for the gyros.

The bias and scale factor of both the accelerometers and gyros can be calculated using the following equations [9]:

(1) $$b=\frac{y_{up}+y_{down}}{2}$$

(2) $$K=\frac{y_{up}-y_{down}-2\times F}{2\times F}$$

where $y_{up}$ is the average measured specific force or angular rate when the sensitive axis is pointed upwards, $y_{down}$ is the average measured specific force or angular rate when the sensitive axis is pointed downwards, and *F* is the known reference signal. F is the magnitude of the local gravity g for the accelerometers, while for the gyroscopes, F is the magnitude of the Earth rotation rate projection to the vertical axis at a given latitude $\omega_{e}$.

However, unlike high-grade gyroscopes, low-grade ones such consumer-grade MEMS suffer from bias instability and noise levels that can completely mask the Earth’s reference signal. Therefore, the Earth rotation can typically only be used for high-grade gyroscopes. In our case, the Six-Position Static test was performed only for the accelerometers. The accuracy of this method depends on how well the axes are aligned with the vertical axes of the local level frame. This standard calibration method can only be used to determine the bias and scale factors of sensors, but cannot estimate axes misalignments, for which a more complex method is required.

Table 6 reports the bias and scale factor (SF) determined for the five smartphones by carrying out the Six-Position Static test.

In this case, the One Plus 7 Pro resulted in being the one with the largest accelerometer bias, while the Vivo X60 pro was the one with smallest. The Samsung SM-A536V had the smallest scale factor and the iPhone XR had the largest.

### 3.3. Gyro Rate Test

**Figure 5:**
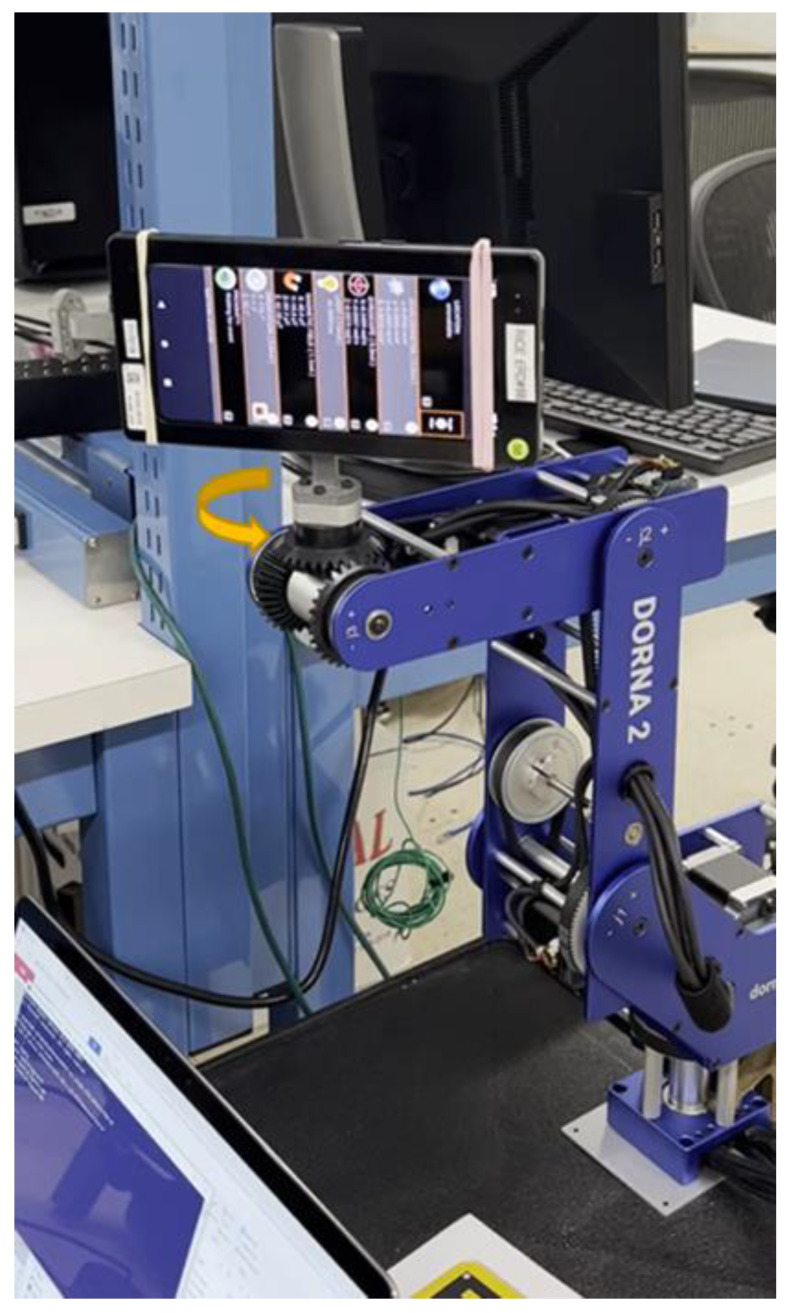
Experimental setup for the Gyro Rate Test showing the robot Dorna 2 and smartphone device.

**Figure 6:**
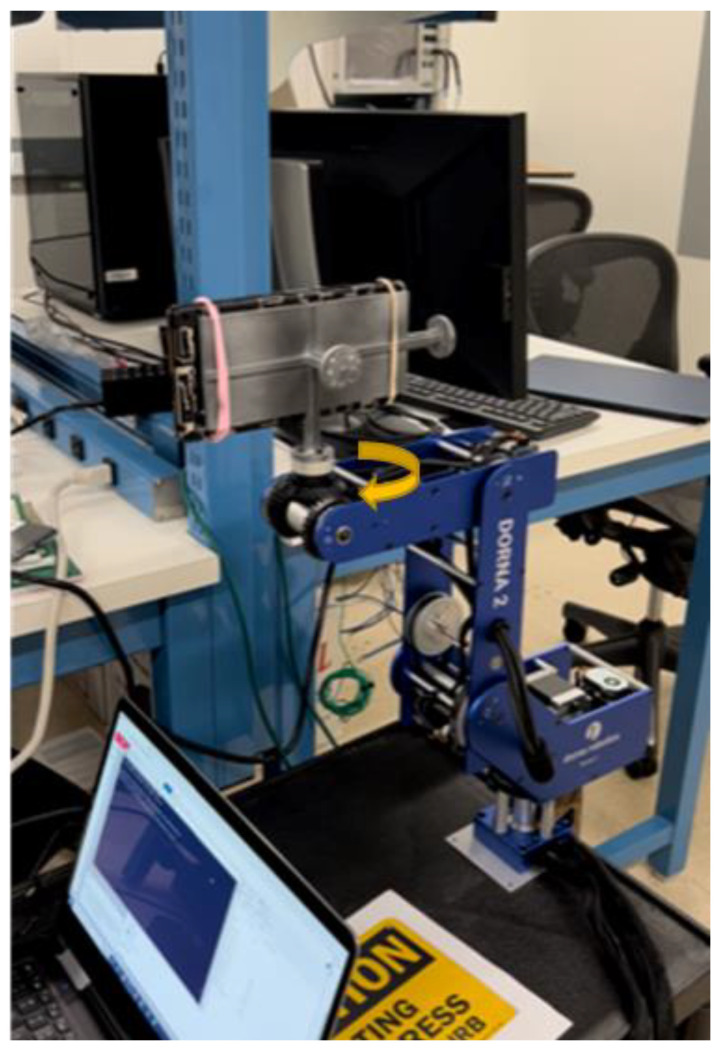
Experimental setup for the Gyro Rate Test showing the robot Dorna 2 and the phone holder.

**Figure 7:**
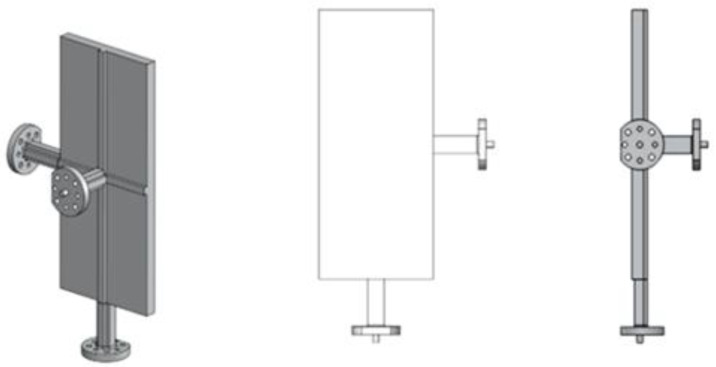
Phone holder that connects the smartphones to the robot head.

In this test, as shown in Figure 5, Figure 6 and Figure 7, we used the robotic arm Dorna 2 [27] and a phone holder we designed and printed, to stimulate each gyro axis with a given reference angular velocity $\omega_{ref}$ and with the same, but in the opposite direction $\omega_{ref-}$.

The output of the gyros was recorded for a few minutes. Therefore, the bias and scale factor were calculated as follows:

(3) $$b=\frac{y_{\omega_{ref}}+y_{\omega_{ref-}}}{2}$$

(4) $$K=\frac{y_{\omega_{ref}}-y_{\omega_{ref-}}-2\times \omega_{ref}}{2\times \omega_{ref}}$$

In general, the test can be repeated with different reference values $\omega_{ref}$ in order to characterize the scale factor and bias as a function of the angular rate. For this paper, we collected data for two representative angular rate values of 10 deg/s and 40 deg/s. Note that the test can also be repeated for different g values for the characterization of the g-dependent errors. In our tests, each gyro axis was aligned with the local vertical axis.

Table 7 and Table 8 report the bias and SF values at 10 deg/s and 40 deg/s, respectively, for all five smartphones.

For the gyros at 10 deg/s, the One Plus 7 Pro exhibited the largest bias and SF values, while the Pixel 7 Pro showed the lowest bias and second-lowest scale factor. The same cannot be said for the gyro bias and SF at 40 deg/s, for which the One Plus 7 Pro showed the highest scale factor but the lowest bias, while the Pixel 7 Pro and Vivo X60 Pro had the highest bias and lowest scale factor, respectively.

## 4. Effect of MEMS Errors on Positioning

The inertial navigation state solution, including position, velocity, and attitude, is affected by errors from three sources: errors in accelerometer and gyro sensor measurements, errors in the initialized state, and processing errors. As discussed in Section 2, measurement errors consist of a stochastic, random component and a deterministic, systematic one. Random noise from accelerometers and gyros has a cumulative effect on navigation errors. Accelerometer and gyro biases are integrated into the navigation equations, producing position, velocity, and attitude errors that increase over time. Attitude errors also affect velocity and position errors. Velocity initialization errors produce a growing position error when integrated.

Processing errors arise due to several factors, including the discretization of the navigation equation, the effects of finite iteration rates, approximations in the gravity model, computational rounding errors, and timing errors [13,15].

Inertial error propagation is also influenced by the host vehicle trajectory; indeed, for example, the coupling of heading errors into position and velocity, as well as the effect of the scale factor and cross-coupling errors, all strongly depend on the host vehicle dynamics [28].

Very simple models of gravity, function of latitude and height only, can be a significant source of error (up to 0.1 mg), particularly if high-precision inertial sensors are used. Timing errors (due to a non-perfect clock within the inertial navigation processor) are generally negligible compared to the other sources.

### 4.1. Short-Term Straight-Line Pure Inertial Propagation

In order to analyze the effect of inertial error propagation, we can evaluate the short-term error propagation given the noise SD and bias values determined in Section 3 and some errors in the initial position, velocity, and attitude states.

Let us consider the simplest case of a host vehicle traveling in a straight and level line at a constant velocity and constant temperature, and derive each error contribution as done in [13]. The effects of the curvature and rotation of the Earth and gravity approximations can be neglected. The error of the position of the INS body frame b, with the respect to the origin of a frame β resolved about the axes of a frame γ error, can be formulated as the integral of the velocity error with a constant velocity error,

(5) $$\delta r_{\beta b}^{\gamma }=\delta v_{\beta b}^{\gamma }t.$$

where t is the integration time, while the velocity error is the integral of the acceleration error with a constant accelerometer bias $b_{a}$,

(6) $$\delta v_{\beta b}^{\gamma }=C_{b}^{\gamma }b_{a}t.$$

where $C_{b}^{\gamma }$ is a rotation matric from frame b to frame γ.

Therefore, the position error will be:

(7) $$\delta r_{\beta b}^{\gamma }=\frac{1}{2}C_{b}^{\gamma }b_{a}t^{2}$$

Note that there is no error propagation between the axes, since we assume that the attitude remains constant.

With a small-angle approximation, the attitude error can be expressed as a vector resolved about a certain set of axes. We can denote with ${\tilde{f}}_{ib}^{b}$ the specific force measured by the accelerometer, and with $\delta \Psi_{\gamma b}^{\gamma }$, the error in the INS-indicated attitude of frame b with respect to a frame γ, resolved about the frame γ axes.

An acceleration error due to the constant attitude error $\delta \Psi_{\gamma b}^{\gamma }$ under a small-angle assumption is:

(8) $$\delta a_{\beta b}^{\gamma }\approx \delta \Psi_{\gamma b}^{\gamma }\times \left(C_{b}^{\gamma }{\tilde{f}}_{ib}^{b}\right)=C_{b}^{\gamma }\left(\delta \Psi_{\gamma b}^{\gamma }\times {\tilde{f}}_{ib}^{b}\right)$$

Velocity and position errors due to an attitude error are:

(9) $$\delta v_{\beta b}^{\gamma }\approx \delta \Psi_{\gamma b}^{\gamma }\times \left[C_{b}^{\gamma }\left(\begin{array}{c} 0\\ 0\\ -g \end{array}\right)\right]t\;\;\;\;\;=C_{b}^{\gamma }\left[\delta \Psi_{\gamma b}^{\gamma }\times \left(\begin{array}{c} 0\\ 0\\ -g \end{array}\right)\right]t\;\;\delta r_{\beta b}^{\gamma }\approx \frac{1}{2}\delta \Psi_{\gamma b}^{\gamma }\times \left[C_{b}^{\gamma }\left(\begin{array}{c} 0\\ 0\\ -g \end{array}\right)\right]t^{2}\;\;\;\;=\frac{1}{2}C_{b}^{\gamma }\left[\delta \Psi_{\gamma b}^{\gamma }\times \left(\begin{array}{c} 0\\ 0\\ -g \end{array}\right)\right]t^{2}$$

An attitude error due to a gyro bias is simply:

(10) $$\delta \Psi_{ib}^{b}\approx b_{g}t$$

Therefore, velocity and position errors due to a gyro bias are:

(11) $$\delta v_{\beta b}^{\gamma }\approx \frac{1}{2}C_{b}^{\gamma }\left[b_{g}\times \left(\begin{array}{c} 0\\ 0\\ -g \end{array}\right)\right]t^{2}\;\delta r_{\beta b}^{\gamma }\approx \frac{1}{6}C_{b}^{\gamma }\left[b_{g}\times \left(\begin{array}{c} 0\\ 0\\ -g \end{array}\right)\right]t^{3}$$

Assuming white noise and denoting the single-sided accelerometer and gyroscope noise PSDs as $S_{a}$ and $S_{g}$, the SDs of position and velocity errors due to accelerometer noise for each axis i∈x,y,z are [13]:

(12) $$\sigma \left(\delta v_{\beta b,i}^{\gamma }\right)=\sqrt{S_{a}t}\;\sigma \left(\delta r_{\beta b,i}^{\gamma }\right)=\sqrt{\frac{1}{3}S_{a}t^{3}}$$

While the SDs of attitude, horizontal position, and horizontal velocity errors due to gyroscope noise are [13]:

(13) $$\sigma \left(\delta \Psi_{\beta b,i}^{\gamma }\right)=\sqrt{S_{g}t}\;\sigma \left(\delta v_{\beta b,j}^{n}\right)=g\sqrt{\frac{1}{3}S_{g}t^{3}}\;\sigma \left(\delta r_{\beta b,j}^{\gamma }\right)=g\sqrt{\frac{1}{5}S_{g}t^{5}}$$

where i∈x,y,z and j ∈North, East.

In a nutshell, according to [13], short-term position error growth due to different sources can be summarized as in Table 9.

**Figure 8:**
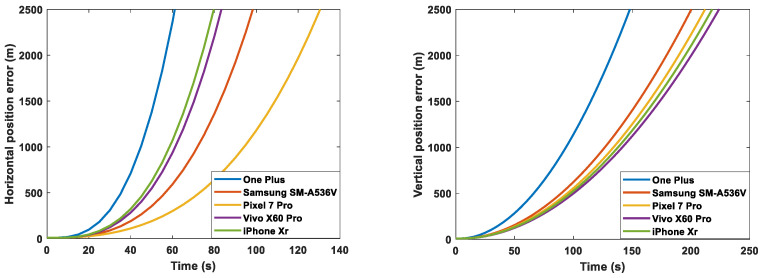
Short-term straight-line position error SD growth per axis for One Plus, iPhone XR, Vivo X60 Pro, Pixel 7 Pro, and Samsung SM-A536V.

**Figure 9:**
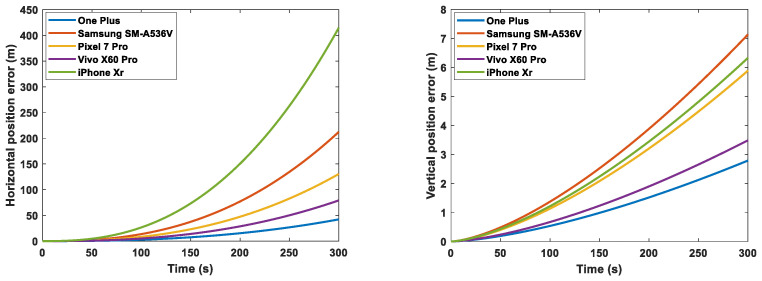
Short-term straight-line position error SD growth per axis for One Plus, iPhone XR, Vivo X60 Pro, Pixel 7 Pro, and Samsung SM-A536V with perfect initial conditions, zero biases, and only noise effect.

For the five smartphones considered, the following figures show the short-term straight-line position error SD growth per axis due to the noise and bias values determined in Section 3, with an initial position error of 10 m, initial velocity error of 0.1 m/s, and initial attitude error of 0.01 rad. The figure on the left displays the horizontal error, while the one on the right illustrates the vertical error. In particular, Figure 8 shows the total error accounting for bias, noise, and errors in the initial condition, while Figure 9 shows the error due to the noise contribution only. We can see that the largest horizontal position error in Figure 8 is accumulated with the MEMS errors of the One Plus 7 Pro, while the smallest is with the ones of the Pixel 7 Pro. This is expected, since the MEMS measurements of the One Plus 7 Pro had the highest accelerometer and gyro bias values, while the ones of the Pixel 7 Pro had the lowest gyro bias. It is interesting to see in Figure 9 that, if noise was the only source of error, the One Plus would outperform, since it showed the lowest noise SD values, but as shown in Figure 8, because of the strongest impact of its large accelerometer and gyro biases, it accumulated the largest position error.

As expected, the gyro biases had the strongest impact on positioning over time. Indeed, this grows with $t^{3}$. Accelerometer biases produce an error in position that grows with $t^{2}$. Approximately, for 2D navigation, uncompensated gyro and accelerometer biases result in position errors of $\frac{1}{6}b_{g}gt^{3}$ and $\frac{1}{2}b_{a}gt^{2}$, respectively [29]. In less than 100 s, a pure inertial propagation using the inertial measurements with the errors found in Section 3 for all the considered smartphones would result in an error of larger than 1 km.

These results only show the position, velocity, and attitude errors when purely propagating the MEMS measurements (pure inertial propagation). In addition to the quality of the MEMS measurements, the final navigation performance of each smartphone also depends on the GNSS measurements; indeed MEMS inertial measurements are usually corrected with GNSS measurements (these may be may be more accurate for one of the considered devices and less for another one). The next section investigates the impact of different MEMS INS measurements with different errors on an INS-GNSS position solution.

### 4.2. INS-GNSS Positioning

In most cases, the MEMS measurements of a smartphones are fused with GNSS observations. If at least four GNSS satellites are available, processing their signals and observations can provide a position solution that can prevent the error drift showed in Figure 8 and Figure 9 of a pure inertial solution. We evaluated the impact of different MEMS measurement errors on an INS-GNSS tightly coupled navigation solution for a short drive of 5 min around our campus in San Jose, California.

We modelled the accelerometer and gyro measurements as follows, according to [13,30], taking into account the mean value across the x,y,z axes of the error components estimated in our lab tests and reported in Section 3, for each of the five considered smartphones.

(14) $${\tilde{f}}_{ib}^{b}=b_{a}+s_{a}f_{ib}^{b}+w_{a}$$

(15) $${\tilde{\omega }}_{ib}^{b}=b_{g}+s_{g}\omega_{ib}^{b}+w_{g}$$

where ${\tilde{f}}_{ib}^{b}$ and ${\tilde{\omega }}_{ib}^{b}$ are the IMU-output specific force and angular rate measurements, $f_{ib}^{b}$ and $\omega_{ib}^{b}$ are the true counterparts, $b_{a}$ and $b_{g}$ are their biases, $s_{a}$ and $s_{g}$ are their scale factors, and $w_{a}$ and $w_{g}$ are their noise, respectively.

GNSS pseudorange ρ and pseudorange rate $\dot{\rho }$ observations were modelled as in Equations (16) and (17), respectively, according to [31,32], by assuming a GPS constellation of 30 satellites and accounting for a signal in space error SD of 1 m, an atmosphere residual error (after applied correction) SD of 2 m for the ionosphere and 0.2 m for the troposphere, a code tracking error SD of 1 m, a range rate tracking error SD of 0.02 m/s, an initial receiver clock offset of 10,000 m, and initial clock drift of 100 m/s.

(16) $$\rho_{i}=\sqrt{{\left(x_{sat}{}_{i}-x_{u}\right)}^{2}+{\left(y_{sat}{}_{i}-y_{u}\right)}^{2}+{\left(z_{sat}{}_{i}-z_{u}\right)}^{2}}+b+errors_{\rho }{}_{i}$$

(17) $${\dot{\rho }}_{i}=\left(v_{{sat}_{i}}-v_{u}\right)\cdot a_{i}+\dot{b}+errors_{{\dot{\rho }}_{i}}$$

In Equation (16), ${\left[\begin{array}{ccc} x_{{sat}_{i}} & y_{{sat}_{i}} & z_{{sat}_{i}} \end{array}\right]}^{T}$ denotes the position vector of the *ith* GPS satellite that is transmitting the signal, ${\left[\begin{array}{ccc} x_{u} & y_{u} & z_{u} \end{array}\right]}^{T}$ is the user’s position vector, and b is the receiver’s clock offset in meters. In Equation (17), $v_{{sat}_{i}}$ and $v_{u}$ are, respectively, the velocity vector of the *ith* transmitting GPS satellite and of the user, $\dot{b}$ represents the clock’s drift expressed as range-rate bias (in m/s), and $a_{i}$ is the line-of-sight (LOS) vector from the user to the *ith* GPS satellite.

**Figure 10:**
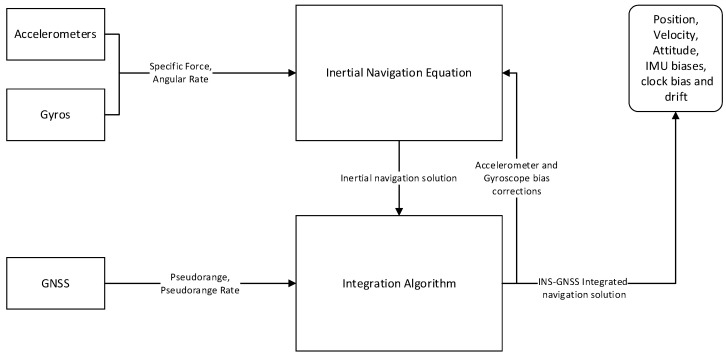
Functional architecture of the adopted INS-GNSS integration.

We filtered the inertial measurements with the simulated GNSS measurements utilizing an INS-GNSS tight integration EKF based on [13]. Figure 10 illustrates the functional architecture of the adopted INS-GNSS integration.

Table 10 reports the EKF formulation described in [32].

The state vector of the EKF is the following:

(18) $$x={\left[\begin{array}{cc} \delta \varphi & \begin{array}{cc} \delta v & \begin{array}{ccc} \delta r & b_{a} & \begin{array}{ccc} b_{g} & \delta \rho_{c}^{GNSS} & \delta {\dot{\rho }}_{c}^{GNSS} \end{array} \end{array} \end{array} \end{array}\right]}^{T}$$

where,

δφ is the attitude error,

δv is the velocity error,

δr is the position error,

$b_{a}$ are the accelerometer biases,

$b_{g}$ are the gyro biases,

$\delta \rho_{c}^{GNSS}$ is the receiver clock offset,

$\delta {\dot{\rho }}_{c}^{GNSS}$ is the receiver clock drift.

The measurement vector is:

(19) $$z=\left[\begin{array}{c} \rho_{GNSS}\\ {\dot{\rho }}_{GNSS} \end{array}\right]\;$$

where $\rho_{GNSS}$ and ${\dot{\rho }}_{GNSS}$ are the pseudoranges and pseudorange rates of the available GPS satellites, respectively.

The measurement innovation vector includes the differences between the GNSS-measured pseudorange and pseudorange rates and the corresponding values predicted by the corrected inertial navigation solution at the same time of validity, by using the estimated receiver clock offset and drift, and navigation-data-indicated satellite positions and velocities.

The matrices $\Phi_{k-1}$,$\;Q_{k-1}$,$\;R_{k},H_{k}$ were implemented according to Chapter 14 of [13].

We estimated five different trajectories using the same INS-GNSS tight integration filter, identical GNSS observations, and identical initial conditions. However, we modeled different sets of inertial measurements based on the MEMS error estimates from the lab tests in Section 3 for each of the five smartphones. We used a precise navigation system that fused a carrier-phase GNSS and higher-grade inertial measurements to provide a reference trajectory (ground truth) for the drive. We then calculated the error of the INS-GNSS-estimated trajectories using this reference.

**Figure 11:**
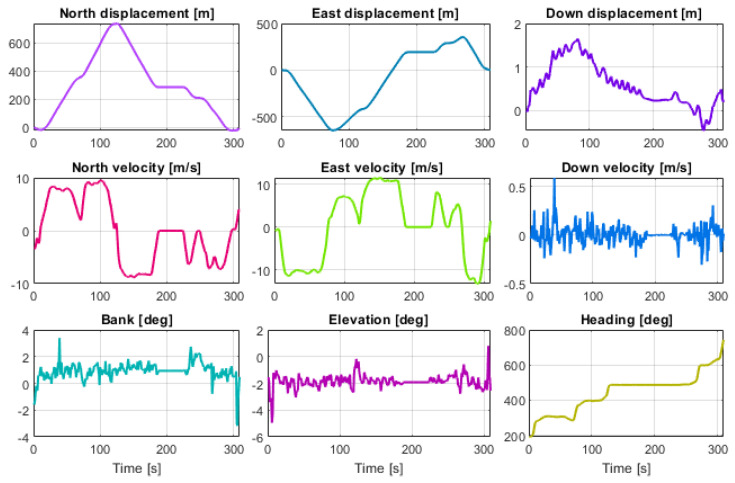
Reference trajectory estimated by the high-precision INS-GNSS navigation system.

Figure 11 shows the motion profile as the North, East, Down (NED) displacements and velocity, bank, elevation, and heading of the reference trajectory. The motion profile includes five turns of approximately 90 deg, with a maximum speed of approximately 10 m/s.

We analyzed two cases: one with a mask angle of 10 deg, corresponding to seven GNSS satellites available for the whole drive, and one with a mask angle of 45 deg, corresponding to a reduced GNSS availability for most of the drive.

**Figure 12:**
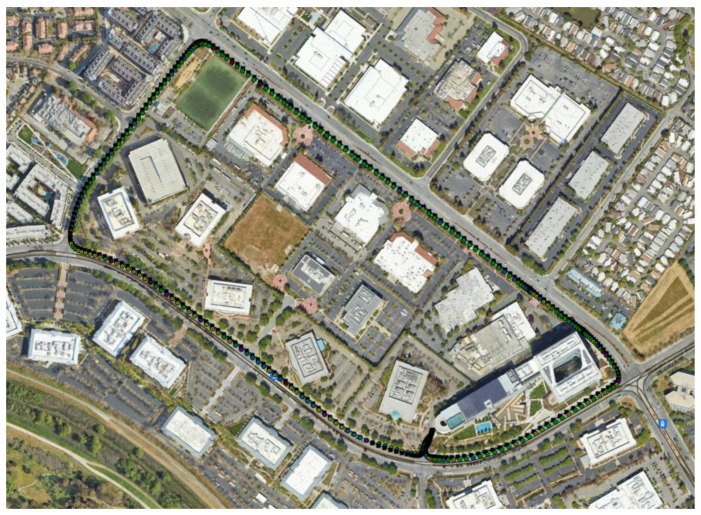
Kml trajectories of reference IMU in black, iPhone XR in green, One Plus 7 Pro in light blue, Vivo X60 Pro in purple, Google Pixel 7 Pro in yellow, and Samsung SM-A536V in red, when assuming a mask angle of 10 deg.

**Figure 13:**
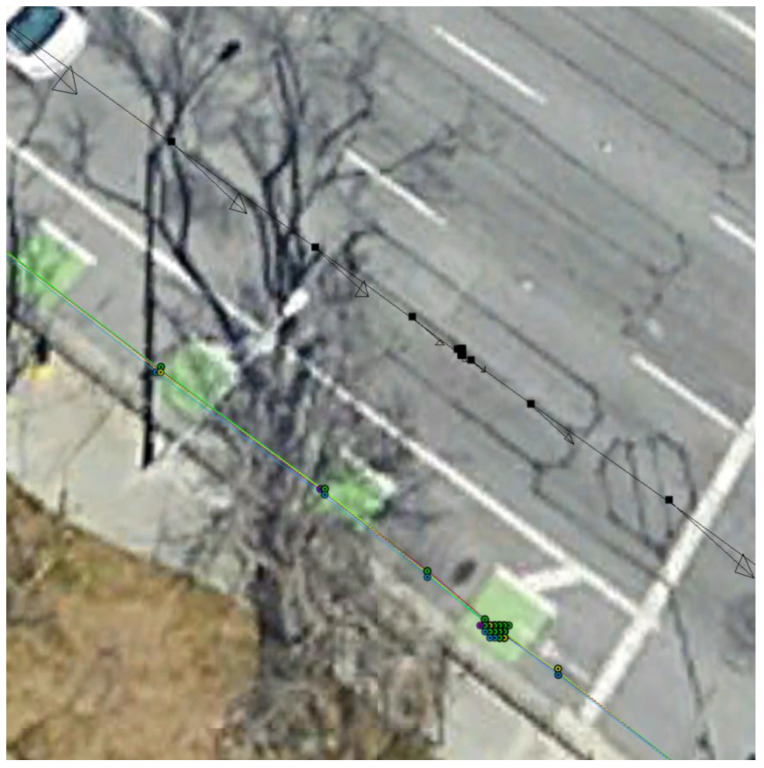
Zoom of Figure 12.

**Figure 14:**
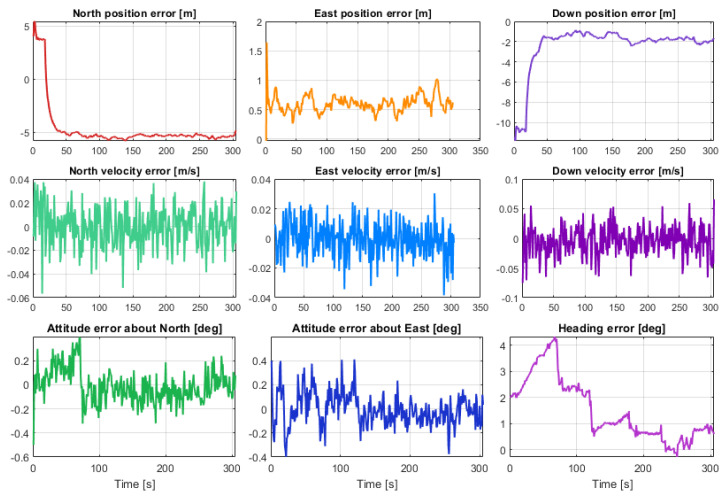
Position, velocity, and attitude estimation errors for Samsung SM-A536V, with a mask angle of 10 deg (corresponding to 7 GNSS satellites available for the whole drive).

**Figure 15:**
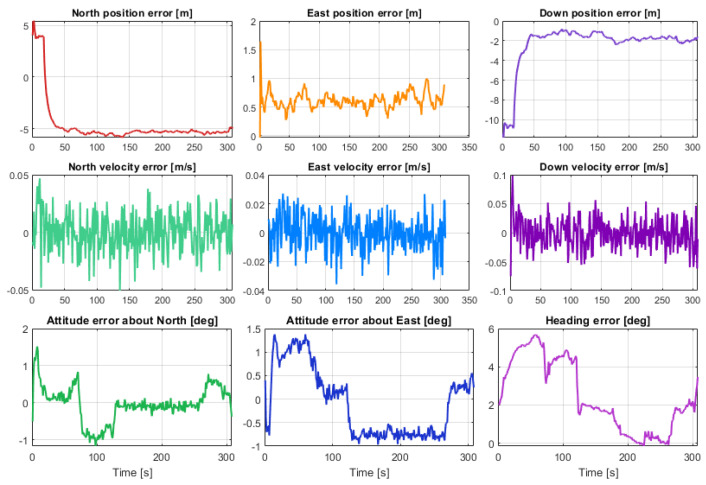
Position, velocity, and attitude estimation errors for One Plus 7 Pro, with a mask angle of 10 deg (corresponding to 7 GNSS satellites available for the whole drive).

Figure 12 shows the kml tracks of the five estimated position solutions obtained with the MEMS measurements of the five considered smartphones, as well as the track of the reference solution, with a mask angle of 10 deg. In general, from Figure 12, there are no evident performance differences. For all the considered smartphones, the GNSS observations from the available seven satellites successfully corrected the inertial solution and prevented any error drift. Small positioning differences can be recognized when zooming into Figure 12, as shown in Figure 13. Some more evident differences in the velocity and especially the attitude estimation errors can be observed by directly comparing the velocity and attitude error components. Figure 14 and Figure 15 show the lowest velocity and attitude estimation errors (obtained with the MEMS errors of the Pixel 7 Pro) and the largest ones (obtained with the MEMS errors of the One Plus 7 Pro), respectively.

**Figure 16:**
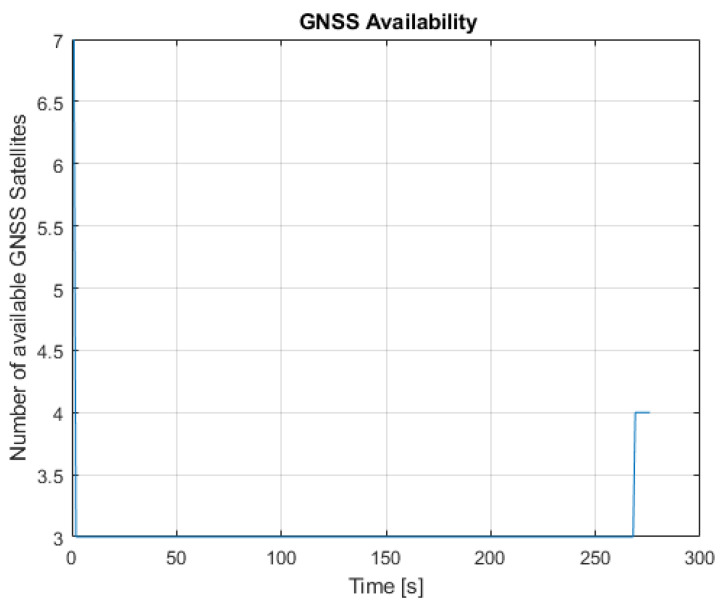
Number of available GNSS satellites with a mask angle of 45 deg.

When increasing the mask angle to 45 deg after initialization, the number of available GNSS satellites dropped to three for the first 268 s, as illustrated in Figure 16.

**Figure 17:**
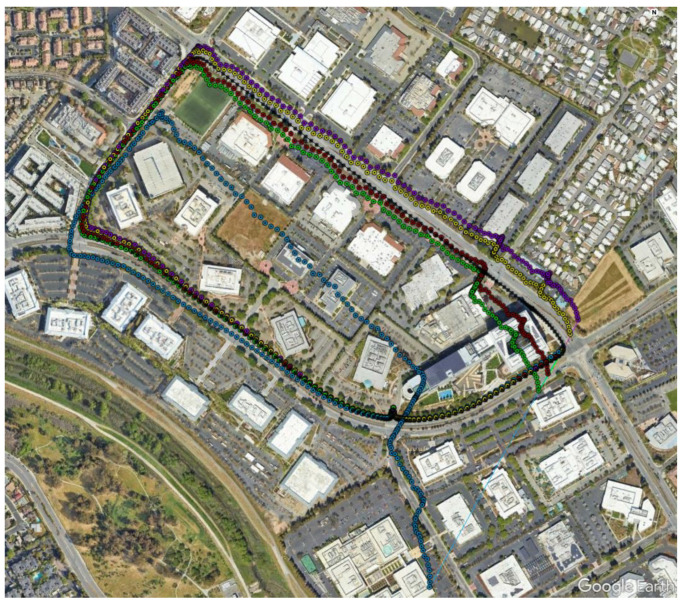
Kml trajectories of reference IMU in black, iPhone XR in green, One Plus 7 Pro in light blue, Vivo X60 Pro in purple, Google Pixel 7 Pro in yellow, and Samsung SM-A536V in red, with a mask angle of 45 deg.

**Figure 18:**
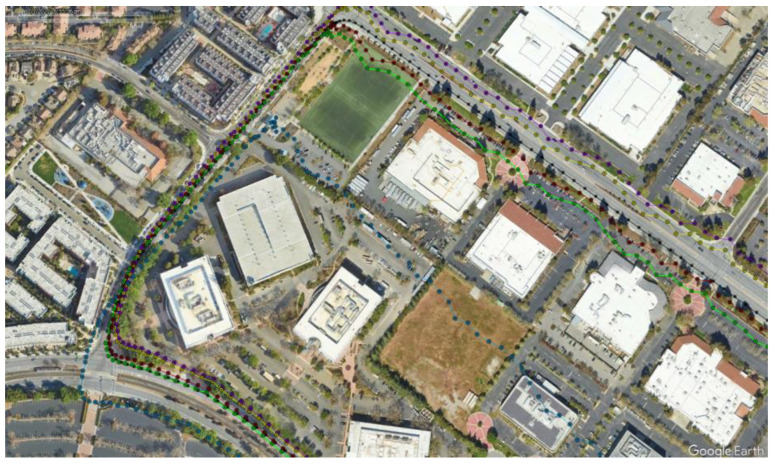
Zoom of Figure 17.

**Figure 19:**
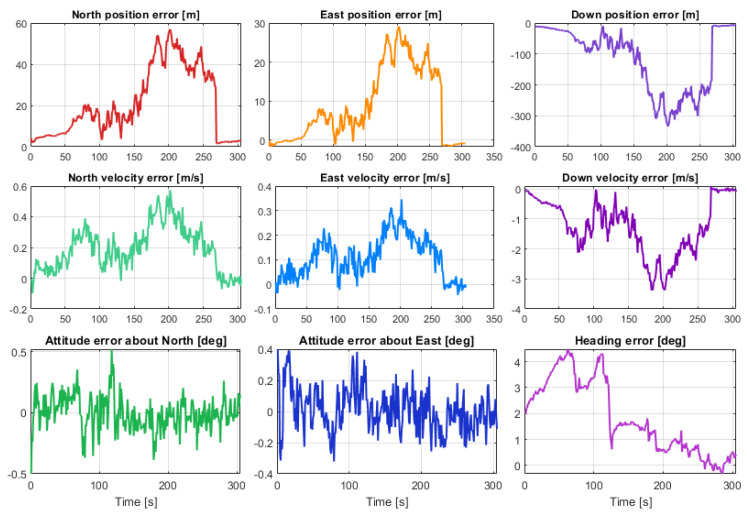
Position, velocity, and attitude estimation errors for Pixel 7 Pro.

**Figure 20:**
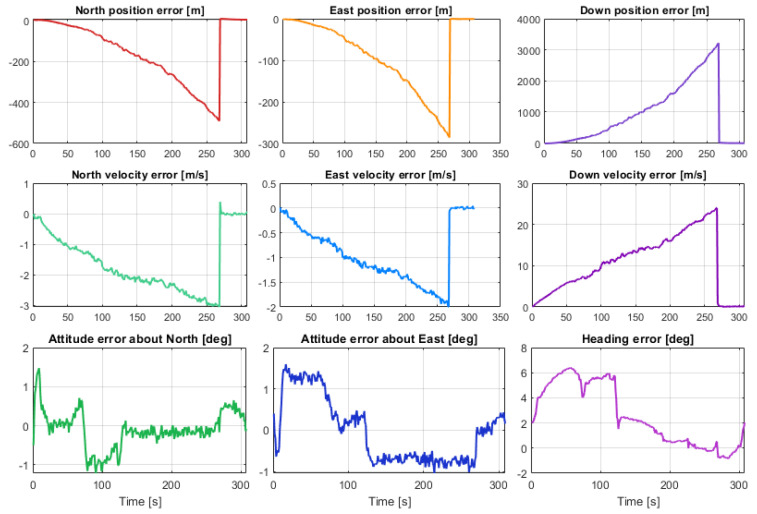
Position, velocity, and attitude estimation errors for One Plus 7 Pro.

Figure 17 and Figure 18 show the same tracks of Figure 12, but obtained with a mask angle of 45 deg. Unlike in Figure 12, where the solutions were obtained with seven available GNSS satellites, for only three GNSS satellites available we can see a substantial degradation of the positioning accuracy with an error drift. In this case, the differences between the performances of the five different smartphones were more evident and relevant. The position solution estimated for the One Plus 7 Pro was the one that diverged first. The one based on the MEMS measurement errors of the Pixel 7 Pro appeared to accumulate the smallest error by the end of the time interval with only three satellites available. Figure 19 and Figure 20 show the position, velocity, and attitude estimation error components obtained for these two smartphones. We can see that the INS-GNSS positioning accuracy in poor GNSS conditions (number of available GNSS satellites less than four) can be substantially different across the five considered smartphones can change substantially. By comparing the North, East, and Down position estimation error components of the Pixel 7 Pro (Figure 19) with those of One Plus 7 Pro (Figure 20), we can observe a difference of about one order of magnitude (e.g., for the Down component, we have errors up to about 320 m for the Pixel 7 Pro and up to about 3200 m for the One Plus 7 Pro).

## 5. Discussion

Section 4.1 and Section 4.2 present an analysis of the short-term effects of the main error sources introduced in Section 2 and determined in Section 3 on the navigation accuracy in two different scenarios and conditions, with corresponding results. While Section 4.1 examined straight-line motion at a constant speed using INS only, Section 4.2 examined motion with five turns of approximately 90 degrees and a maximum speed of approximately 10 m/s using both INS and GNSS. From Section 4.1, we can see that pure inertial navigation using MEMS measurements with a smartphone can be accurate for a very short time (a few seconds) and quickly becomes highly inaccurate. For all five smartphones, the accumulated horizontal error on a short-term straight-line propagation was larger than 1 km in just 100 s. We can also see that phones with larger uncompensated biases had a much faster error growth, leading to significant differences in their performance when navigating in GNSS-denied environments such as tunnels and parking garages. From Section 4.2, we can see that, as long as more than four GNSS satellites are available, MEMS measurements from a phone with larger biases and higher noise did not lead to significantly larger navigation errors than those with lower biases and lower noise, unlike in the case of the pure inertial propagation in Section 4.1. However, there was a non-negligible difference in performance when only three GNSS satellites were available. In this case, the error drifts by more than 3 km within a span of approximately 250 s during a drive that includes three turns, each of approximately 90 deg.

An important aspect to consider is that the accuracy of the final position solution provided by a smartphone, in reality, will also strongly depend on the availability and quality of the GNSS measurements, which here in this paper, were simulated to be identical for each smartphone, in order to isolate and uniquely evaluate the MEMS impacts on positioning performance. Indeed, one smartphone may embed MEMS of a higher quality, but a GNSS receiver with a poorer signal processing performance. In addition, some smartphones may utilize measurements from additional sensors, such as a magnetometer, barometer, motion constraints modelled as virtual measurements, and different estimation algorithms, which can all affect the achievable navigation performance. Therefore, the reader should not solely rely on the results presented in this paper to determine the positioning capabilities of the considered smartphones. How accurate the position solution of one smartphone was with respect to that of another smartphone presented here may not reflect the overall performances, which are based on the individual navigation architecture and on the individual availability and quality of the measurements provided by additional sensors. Nevertheless, the goal of this work was to quantify the main error components affecting MEMS INS measurements in a smartphone and analyze their effect on positioning, rather than determining what smartphone showed the overall best navigation capabilities.

Numerous literature studies have proposed different methods for enhancing the achievable navigation accuracy when using an INS and or the fusion of an INS with a GNSS. Zero updates and motion constraints are techniques that can help to improve the accuracy of position tracking when using IMUs in smartphones and other devices [13,33,34]. These techniques can be combined with machine learning methods and with the processing of the measurements of other sensors to further improve the achievable positioning accuracy [35,36,37]. In particular, Zero velocity updates (ZVUs) are used to maintain the alignment and calibration of an INS when the host vehicle or user is stationary during navigation. This technique is particularly useful in poor GNSS signal environments, such as urban areas, and can be used for land vehicle navigation. Zero angular rate updates (ZARUs) are another technique that can be used to improve the accuracy of position tracking. ZARUs and ZVUs are often performed together, but may also be implemented independently.

Motion constraints are used to correct and calibrate the errors in an INS by taking advantage of the limitations in the movement of a vehicle or pedestrian. These constraints provide additional information to the navigation solution based on the operating context. They are also known as nonholonomic constraints, which introduce a dependency of the state estimates on their previous values [13].

Machine learning methods can be used to improve position tracking, e.g., by identifying periods when IMUs are stopped (zero-velocity detection) and estimating the displacement of the sensors during periods of movement. For example, classifiers such as Random Forest, Support Vector Machines (SVM), and neural networks based on Long Short-Term Memory (LSTM) layers can be used to identify zero-velocity periods [36,37].

In addition, a higher accuracy and robustness can be achieved by combining data from additional available embedded sensors, such as magnetometers, barometers, Wi-Fi systems, and cameras [38,39]. The fusion of data from these sensors can overcome their individual limitations and provide a more precise and reliable navigation solution.

## 6. Conclusions

In this paper, we analyzed the main sources of the stochastic and deterministic errors affecting MEMS sensor measurements in five commercial smartphones. We carried out specific laboratory tests to determine the standard deviations, bias instabilities, random walks, rate random walks, biases, and scale factors of both the accelerometers and gyros in these devices.

Our results in Section 3 showed that the MEMS measurement errors of one smartphone could be significantly larger than those of another. These differences were large enough to result in substantially different INS and INS-GNSS navigation performances.

Some of the MEMS sensors may not have been calibrated before being embedded into their respective smartphones, considering the large biases and scale factors we determined.

In general, the measurements of the MEMS sensors embedded in the smartphones we considered are not accurate enough for meaningful, long-term pure inertial navigation. Fusing MEMS measurements with a GNSS prevents drift, as long as at least four GNSS satellites are available. However, when fusing three GNSS observations only, as expected, the solution will be affected by a drifting error, whose severity will mainly depend on the bias of the inertial measurements.

In our future work, we plan to conduct more specific tests to determine higher-order errors and evaluate their effect on positioning accuracy, also modelling motion constraints and additional measurements from other sensors.

## Figures and Tables

**Table 1:** Tested smartphones and corresponding inertial sensors they integrate.

Smartphone Model	Manufacturer	Accelerometers	Gyroscopes
*Samsung SM-A536V*	SamsungElectronics Co Ltd. (Suwon, Republic of Korea)	LSM6DSOTR	LSM6DSOTR
*Google Pixel 7 Pro*	Google LLC (Mountain View, CA, USA)	LSM6DSV	LSM6DSV
*Vivo X60 Pro*	Vivo Communication Technology Co., Ltd. (Dongguan, China)	LSM6DSO	LSM6DSO
*iPhone XR*	Apple Inc. (Cupertino, CA, USA)	N.A.	N.A.
*One Plus 7 Pro*	OnePlus Technology Co., Ltd. (Shenzhen, China)	BMI160	BMI160

**Table 2:** Formula, curve slope, and coefficient value for each noise parameter [10].

Error Type	log(σ) vs. log(τ)	Curve Slope	Coefficient Value
Random Walk	$\log\left(\sigma \right)=-\frac{1}{2}\log\left(\tau \right)+\log\left(N\right)$	$-\frac{1}{2}$	N=σ(1)
Bias Instability	$\log\left[\sigma \left(f_{0}\right)\right]=\log\left(\sqrt{\frac{2ln2}{\pi }}\cdot B\right)$	0	$B=\frac{\sigma \left(f_{0}\right)}{0.664}$
Rate Random Walk	$\log\left(\sigma \right)=\frac{1}{2}\log\left(\tau \right)+\log\left(\frac{K}{\sqrt{3}}\right)$	$+\frac{1}{2}$	K=σ(3)

**Table 3:** Standard deviation.

		SD Accelerometer [m/s^2^]	SD Gyro [rad/s]
**Samsung SM-A536V**	X	0.010049068	0.001242583
	Y	0.010441081	0.001253576
	Z	0.011421130	0.001586549
	mean	**0.010637093**	0.001360903
**Google Pixel 7 Pro**	X	0.007663586	0.000889025
	Y	0.007970449	0.000907514
	Z	0.010705712	0.000712619
	mean	0.008779916	0.000836386
**Vivo X60 Pro**	X	0.005928450	0.000467233
	Y	0.005631080	0.000652637
	Z	0.004041136	0.000402250
	mean	0.005200222	0.000507373
**iPhone XR**	X	0.009588520	0.001465648
	Y	0.007880521	0.002250331
	Z	0.010838779	0.004264051
	mean	0.009435940	**0.002660010**
**One Plus 7 Pro**	X	0.004113892	0.000273676
	Y	0.002455876	0.000302612
	Z	0.005909729	0.000235906
	mean	**0.004159832**	**0.000270731**

**Table 4:** Accelerometer bias instability and random walks.

		Bias Instability B [m/s^2^]	Velocity Random Walk N [(m/s^2^)/sqrt(Hz)]	Acceleration Random Walk K [(m/s^2^) × sqrt(Hz)]
**Samsung SM-A536V**	X	0.000474035	0.002232118	0.001288714
	Y	0.000516343	0.002174748	0.001255592
	Z	0.003006905	0.018887106	0.010904476
	mean	**0.001332428**	**0.007764657**	**0.004482927**
**Google Pixel 7 Pro**	X	0.000208068	0.001712572	0.000988754
	Y	0.000175490	0.001835399	0.001059668
	Z	0.000268215	0.002325118	0.001342408
	mean	**0.000217258**	0.001957696	0.001130277
**Vivo X60 Pro**	X	0.000418808	0.001049093	0.000605583
	Y	0.000356650	0.001127931	0.000651211
	Z	0.000256732	0.000890106	0.000513903
	mean	0.000344063	**0.001022377**	0.000590232
**iPhone XR**	X	0.000910670	0.001179936	0.000313525
	Y	0.000571580	0.001133789	0.000124237
	Z	0.001166650	0.001751674	0.000050204
	mean	0.000882967	0.001355133	**0.000162655**
**One Plus 7 Pro**	X	0.000285625	0.000534956	0.000308857
	Y	0.000416224	0.000645493	0.000372675
	Z	0.000381871	0.003891451	0.002246731
	mean	0.000361240	0.001690633	0.000976088

**Table 5:** Gyro bias instability and random walks.

		Bias Instability B [rad/s]	Angular Random Walk N [(rad/s)/sqrt(Hz)]	Rate Random Walk K [(rad/s) × sqrt(Hz)]
**Samsung SM-A536V**	X	0.000016138	0.000272514	0.000157336
	Y	0.000009182	0.000370377	0.000213837
	Z	0.000006144	0.000419685	0.000242305
	mean	0.000010488	0.000354192	0.000204493
**Google Pixel 7 Pro**	X	0.000011138	0.000204410	0.000118016
	Y	0.000008738	0.000207131	0.000119587
	Z	0.000008855	0.000159000	0.000091799
	mean	**0.000009567**	**0.000190180**	**0.000109801**
**Vivo X60 pro**	X	0.000170295	0.001684784	0.000972710
	Y	0.000107853	0.001583311	0.000914125
	Z	0.000124753	0.000791048	0.000456712
	mean	**0.000134300**	0.001353048	0.000781182
**iPhone XR**	X	0.000030634	0.000296360	0.000001209
	Y	0.000077489	0.000403337	0.000003604
	Z	0.000162134	0.003658483	0.000004697
	mean	0.000090033	**0.001452726**	**0.000003170**
**One Plus 7 Pro**	X	0.000026131	0.000064993	0.000037523
	Y	0.000013615	0.000324383	0.000187282
	Z	0.000025621	0.000301456	0.000174046
	mean	0.000021789	0.000230277	0.000132950

**Table 6:** Accelerometer bias and scale factor.

		Bias [m/s^2^]	Scale Factor
**Samsung SM-A536V**	X	0.090409789	0.013743845
	Y	0.084114571	−0.000975747
	Z	−0.056371548	−0.005114418
	mean	0.076965303	**0.006611337**
**Google Pixel 7 Pro**	X	−0.023521688	−0.005581130
	Y	0.047652858	−0.002604630
	Z	0.087067622	−0.005120735
	mean	0.052747389	0.004435498
**Vivo X60 Pro**	X	−0.009156655	0.001514275
	Y	0.005098368	0.005985413
	Z	−0.041585923	−0.006538473
	mean	**0.018613649**	0.004679387
**iPhone XR**	X	0.026573000	0.000379000
	Y	0.086776000	0.001399000
	Z	−0.001050000	−0.001690000
	mean	0.038133000	**0.001156000**
**One Plus 7 Pro**	X	−0.224982900	−0.000486660
	Y	0.174106304	0.011129523
	Z	0.219099173	0.002375162
	mean	**0.206062792**	0.004663782

**Table 7:** Gyro bias and scale factor at 10 deg/s.

		Bias [rad/s]	Scale Factor
**Samsung SM-A536V**	X	0.000140756	−0.000124110
	Y	−0.002706500	0.029467986
	Z	0.001828869	0.000059642
	mean	0.001558708	0.009883899
**Google Pixel 7 Pro**	X	−0.000000864	−0.000187850
	Y	0.001881322	−0.001169500
	Z	0.000029721	−0.000121930
	mean	**0.000637295**	0.000493093
**Vivo X60 pro**	X	−0.000132100	−0.000157070
	Y	−0.004326800	−0.000910130
	Z	0.003383160	0.000328004
	mean	0.002614020	**0.000465068**
**iPhone XR**	X	0.002700000	0.004300000
	Y	0.005600000	0.006200000
	Z	0.000686741	0.004902211
	mean	0.002995580	0.005134070
**One Plus 7 Pro**	X	0.003954450	−0.025509810
	Y	0.003646413	−0.025461340
	Z	−0.012241020	−0.028221540
	mean	**0.006613961**	**0.026397563**

**Table 8:** Gyro bias and scale factor at 40 deg/s.

		Bias [rad/s]	Scale Factor
**Samsung SM-A536V**	X	0.000298200	−0.001807200
	Y	0.009179700	−0.042710310
	Z	−0.002227920	0.000059642
	mean	0.003901940	0.014859037
**Google Pixel 7 Pro**	X	−0.002267500	−0.002382100
	Y	−0.017213000	−0.020348950
	Z	−0.000061247	−0.001558800
	mean	**0.006513900**	0.008096617
**Vivo X60 pro**	X	−0.000131400	−0.000153200
	Y	−0.001904000	−0.002162770
	Z	−0.000011560	−0.003430900
	mean	0.000682333	**0.001915623**
**iPhone XR**	X	0.000340000	0.006000000
	Y	0.000830000	0.007300000
	Z	−0.000633130	0.004619750
	mean	0.000601043	0.005973250
**One Plus 7 Pro**	X	0.000037859	−0.123923290
	Y	0.000031763	−0.119939640
	Z	−0.000102620	−0.124866720
	mean	**0.000057440**	**0.122909883**

**Table 9:** Short-term velocity and position error growth.

Error Source	Velocity Error	Position Error	Attitude Error
Initial velocity error, $\delta v_{\beta b}^{\gamma }$	$\delta v_{\beta b}^{\gamma }$	$\delta v_{\beta b}^{\gamma }t$	0
Initial attitude error,$\;\delta \Psi_{\gamma b}^{\gamma }$	$C_{b}^{\gamma }\left[\delta \Psi_{\gamma b}^{\gamma }\times \left(\begin{array}{c} 0\\ 0\\ -g \end{array}\right)\right]t$	$\frac{1}{2}C_{b}^{\gamma }\left[\delta \Psi_{\gamma b}^{\gamma }\times \left(\begin{array}{c} 0\\ 0\\ -g \end{array}\right)\right]t^{2}$	$\delta \Psi_{\gamma b}^{\gamma }$
Accelerometer bias,$\;C_{b}^{\gamma }b_{a}$	$C_{b}^{\gamma }b_{a}t$	$\frac{1}{2}C_{b}^{\gamma }b_{a}t^{2}$	0
Gyro bias,$\;b_{g}$	$\frac{1}{2}C_{b}^{\gamma }\left[b_{g}\times \left(\begin{array}{c} 0\\ 0\\ -g \end{array}\right)\right]t^{2}$	$\frac{1}{6}C_{b}^{\gamma }\left[b_{g}\times \left(\begin{array}{c} 0\\ 0\\ -g \end{array}\right)\right]t^{3}$	$b_{g}t$
Accelerometer noise,$\;S_{a}$	$\sqrt{S_{a}t}$	$\sqrt{\frac{1}{3}S_{a}t^{3}}$	0
Gyro noise,$\;S_{g}$	$g\sqrt{\frac{1}{3}S_{g}t^{3}}$	$g\sqrt{\frac{1}{5}S_{g}t^{5}}$	$\sqrt{S_{g}t}$

**Table 10:** Adopted EKF formulation [32].

Quantity	Formulation
Predicted state vector	${\hat{x}}_{k}^{-}={\hat{x}}_{k-1}^{+}+\int_{k-1}^{k}f\left(x,t\right)\;dt$
Predicted system noise covariance matrix	$P_{k}^{-}=\Phi_{k-1}P_{k-1}^{+}\Phi_{k-1}^{T}+Q_{k-1}$
Kalman Gain matrix	$K_{k}=P_{k}^{-}H_{k}^{T}{\left(H_{k}P_{k}^{-}H_{k}^{T}+R_{k}\right)}^{-1}$
Corrected state estimate	${\hat{x}}_{k}^{+}={\hat{x}}_{k}^{-}+K_{k}\left(z_{k}-h\left({\hat{x}}_{k}^{-}\right)\right)\;\;\;\;\;\;\;\;={\hat{x}}_{k}^{-}+K_{k}\delta z_{k}^{-}$
Corrected system noise covariance matrix (Joseph form)	$P_{k}^{+}=\left(I-K_{k}H_{k}\right)P_{k}^{-}{\left(I-K_{k}H_{k}\right)}^{T}\;\;\;\;\;\;\;\;+K_{k}R_{k}K_{k}^{T}$

## Data Availability

Not applicable.
